# Locus-specific DNA methylation of *Mecp2* promoter leads to autism-like phenotypes in mice

**DOI:** 10.1038/s41419-020-2290-x

**Published:** 2020-02-03

**Authors:** Zongyang Lu, Zhen Liu, Wei Mao, Xinying Wang, Xiaoguo Zheng, Shanshan Chen, Beibei Cao, Shisheng Huang, Xuliang Zhang, Tao Zhou, Yu Zhang, Xingxu Huang, Qiang Sun, Jia-Da Li

**Affiliations:** 1 School of Life Science and Technology, ShanghaiTech University, 100 Haike Rd., Pudong New Area, Shanghai, 201210 China; 2 CAS Center for Excellence in Molecular Cell Science, Shanghai Institute of Biochemistry and Cell Biology, Chinese Academy of Sciences, University of Chinese Academy of Sciences, 320 Yueyang Road, Shanghai, 200031 China; 3 University of Chinese Academy of Sciences, 100049 Beijing, China; 4 Institute of Neuroscience, Chinese Academy of Sciences (CAS) Key Laboratory of Primate Neurobiology, CAS Center for Excellence in Brain Science and Intelligence Technology, Shanghai Institutes for Biological Sciences, CAS, Shanghai, 200031 China; 5 Hunan Key Laboratory of Animal Models for Human Genetics, School of Life Sciences, Central South University, 110 Xiangya Road, Changsha, 410078 China

**Keywords:** Autism spectrum disorders, Epigenetics and behaviour

## Abstract

Autism spectrum disorder (ASD) is a neurodevelopmental disease with a strong heritability, but recent evidence suggests that epigenetic dysregulation may also contribute to the pathogenesis of ASD. Especially, increased methylation at the *MECP2* promoter and decreased *MECP2* expression were observed in the brains of ASD patients. However, the causative relationship of *MECP2* promoter methylation and ASD has not been established. In this study, we achieved locus-specific methylation at the transcription start site (TSS) of *Mecp2* in Neuro-2a cells and in mice, using nuclease-deactivated Cas9 (dCas9) fused with DNA methyltransferase catalytic domains, together with five locus-targeting sgRNAs. This locus-specific epigenetic modification led to a reduced *Mecp2* expression and a series of behavioral alterations in mice, including reduced social interaction, increased grooming, enhanced anxiety/depression, and poor performance in memory tasks. We further found that specifically increasing the *Mecp2* promoter methylation in the hippocampus was sufficient to induce most of the behavioral changes. Our finding therefore demonstrated for the first time the casual relationship between locus-specific DNA methylation and diseases symptoms in vivo, warranting potential therapeutic application of epigenetic editing.

## Introduction

Autism spectrum disorder (ASD) represents a group of neurodevelopmental diseases characterized by defective social interaction and stereotypic behaviors. It is highly heritable, but has great genetic and clinical heterogeneity. More than 700 risk genes have been identified for ASD, but the effect of each gene is quite small for the great majority of them^1,2^. Accumulating evidence suggests that epigenetic factors may also play an important role in ASD pathogenesis^3^. For instance, there is considerable discordance in ASD-related behaviors between the monozygotic twins^4^. Furthermore, epigenetic modification involving DNA methylation has been identified in ASD patients for several ASD-associated genes, such as *MECP2*, *FMR1*, and *SHANK3*^5–10^.

The gene *MECP2* encodes methylated CpG-binding protein 2 (MeCP2), a putative transcriptional repressor that binds to methylated CpGs. Loss of *MECP2* results in Rett syndrome (RTT)^11^, and patients with RTT exhibit a broad range of impairment in social behaviors, cognition, and coordination. Recently, mutations in *MECP2* have also been identified in sporadic ASD patients^12^. Furthermore, increased *MECP2* promotor methylation and decreased *MECP2* expression were observed in the brain of ASD patients^7,8^, but their causative role in the ASD pathogenesis remains to be clarified.

In this study, we examined the role of *Mecp2* promoter methylation in ASD pathogenesis by using an epigenetic editing strategy. First, we developed an epigenome-editing approach using nuclease-deactivated Cas9 (dCas9) fused with DNA methyltransferase catalytic domains, together with five locus-targeting sgRNAs. We then demonstrated its reliability in specifically increasing the methylation at *Mecp2* promoter using Neuro-2a cell cultures. We further generated mice with specific methylation at this TSS and demonstrated that its methylation down-regulated *Mecp2* expression and induced autism-like behaviors. Finally, we found that specifically increasing the *Mecp2* methylation in the hippocampus is able to induce most of the behavioral alteration.

## Materials and methods

### Animals

All the experimental procedures were approved by the Institutional Animal Care and Use Committee (IACUC) of School of Life Sciences of Central South University, Changsha, China.

### Plasmid design and construction

The pST1374-CMV-Cas9-NLS plasmid (Addgene, 44758) was used as the backbone for construction. Briefly, the CMV promotor was replaced by an EF1α promotor at first. In order to generate DNMT3L-DNMT3A single-chain fusion protein, we have introduced the C-terminal domain of human DNMT3L (amino acids M178-S380) to the N-terminus of the catalytic domain of human DNMT3A (amino acids P627-V912), with a 16-amino acid linker (SSGRSFSSGLVPRGSH). The DNMT3L-DNMT3A was then fused to the N-terminus of dead Cas9 (dCas9). Dead DNMT3A mutation (C710S in human DNMT3A) and dead Cas9 mutation (D10A, H840A, and N863A in Cas9) were introduced using Mut-Express II Fast Mutagenesis Kit V2 (Vazyme) following manufacturer’s instructions. The oligonucleotides for sgRNAs were synthesized, annealed, and cloned into the Bsa I site in the pGL3-sgRNA vector as described previously^13,14^. For AAV-based methylation vectors, the same sequence of DNMT3A was fused to the N-terminus of dCas9, and dCas9 was split into two parts between amino acids E573 and C574. The sequences for primers were listed in Table S3.

### Cell culture and transfection

Neuro-2a (N2a) cells (from male mice) were purchased from ATCC (ATCC, CCL-131) without mycoplasma contamination and cultured in DMEM containing 10% FBS in 37 °C under 5% CO_2_ atmosphere. Twenty six cells were transfected using Lipofectamine 2000 reagent (Life Technologies) according to manufacturer’s protocols.

### T7ENI cleavage assay and sequencing

Cells were harvested with lysis buffer (10 μM Tris-HCl, 0.4 M NaCl, 2 μM EDTA, 1% SDS and 100 μg/ml Proteinase K) at 55 °C for 6 h. Genomic DNA was extracted by phenol-chloroform and isopropyl alcohol precipitation according to standard procedures.

The editing efficiency was estimated by T7 endonuclease I assay. Briefly, the TSS region of *Mecp2* were PCR-amplified from genomic DNA and the products were purified by cleanup kit (Axygen) according to the manufacturer’s protocol. The sequences for primers were listed in Table S3. Purified PCR products were denatured in NEB buffer2 (NEB), and then digested with T7ENI (Vazyme) for 30 min. Digested PCR products were resolved on a 2% agarose gel. PCR-amplified fragments were also ligated into pMD19T vector (Takara). After transformation, colonies with ligation products were sequenced for analyzing the editing efficiency.

### Immunofluorescent staining

Mice were trans-cardially perfused with 4% paraformaldehyde (PFA), and brains were dissected into 4% PFA for post-fixation overnight. Following dehydration in 30% sucrose, brains were sectioned at 30 µm and subjected to immunofluorescence staining. Briefly, floating cryosections were washed in PBS, and then incubated overnight with primary antibody (rabbit anti-MeCP2, 1:1000; cell signaling, 3456 S) diluted in blocking buffer (1.5% BSA, 0.1% Triton-X, 5% NGS, pH7.4, 1 × PBS). After three washes in PBST (0.1% Triton-X, pH7.4, 1 × PBS), sections were incubated for 2 h with the secondary antibody (594-AffiniPure Donkey Anti-Rabbit IgG (H + L), 1:1000; Jackson, 711–585–152). Hoechst 33258 (1:5000; Yeasen) was used for nuclei staining. After three washes in PBST, samples were mounted on the glass slices. Images were captured using a Zeiss Axioimager Z2 microscope and processed using the Image J Fiji software.

### Western blot

Brains were dissected into ice-cold PBS, the hippocampus and parietal cortex were rapidly dissected out. Tissue samples were lysed with 500 μl ice-cold RIPA buffer (Beyotime Biotechnology) containing 1 mM protease inhibitor PMSF (Beyotime Biotechnology) and PIC (Sigma). Cell lysates were homogenized by a homogenizer (PRO Scientific, Inc.) and the protein concentration was determined using a BCA Protein Assay Kit (Thermofisher, 23227). Protein lysates were dissolved in SDS-PAGE protein loading buffer and heated at 95 ^o^C for 10 min. Samples were separated on 10% SDS-PAGE gels (Epizyme, Lnc) and transferred onto PVDF membranes (Immobillon). After blocking with TBST solution containing 5% skimmed milk for 1 h at room temperature, membranes were incubated with primary antibodies (rabbit anti-MeCP2, 1:2000; cell signaling, 3456S; mouse anti-Actin, 1:5000; Sigma, A4700–2ML) followed by HRP-conjugated secondary anti-rabbit and anti-mouse antibodies (1:5000; Vector lab). Blots were imaged with Amersham Imager 600 and quantified using the Image J Fiji software.

### RT-qPCR

Total RNA was prepared from cultured cells or tissues using TransZol Up Plus RNA Kit (TransGen). RNA was converted to cDNA using RT Master Mix (Takara, RR036A). Gene expression levels were measured with ViiA7 Real-Time PCR System (Applied Biosystems) using SYBR qPCR Master Mix (Vazyme) according to the manufacturer’s instructions. Primer information was described in Table S3.

### Mouse zygote injection and embryo transfer

Female C57BL/6 mice (4-week-old) were super-ovulated and mated to C57BL/6 male mice. Zygotes were collected from oviducts of the female mice. For zygotes injection, well mixed plasmids (25 ng/μl 5U6 sgRNAs + 50 ng/μl DNMT3L-DNMT3A-dCas9 or 50 ng/μl DNMT3L-DNMT3A-mut- dCas9) were injected into the cytoplasm of zygotes in a droplet of M2 medium containing 5 µg/ml cytochalasin B (CB) using a piezo (Primetech) microinjector. The injected zygotes were cultured in KSOM medium at 37 °C under 5% CO_2_ and transferred to oviducts of pseudopregnant ICR females at 0.5 dpc.

### Behavior test overview

Male mice at the age of 3–18 weeks were used for all tests. The mice were transferred to the animal facility 2 weeks before the behavioral tests. Mice was allowed to habituate to the testing room for at least 30 min. Three chamber, EPM, open field tests were measured by automated software. STFP, novel objective recognition, tail suspension, and self-grooming tests were scored manually.

### Social interaction tests

Three-chamber test: The three-chamber test was performed as described previously^15^. The apparatus was comprised of a rectangular box (70 cm × 36 cm × 30 cm) divided into three chambers with partitions. The test mouse was placed in the closed middle chamber to habituate for 5 min. Each side chamber contained an empty wire cage. After habituation, one stranger mouse (stranger 1, 10–12-week-old male) was introduced to the wire cage on one side. Another empty cage contained an inanimate object. The test mouse was allowed to explore the entire apparatus for 10 min, and time spent in each chamber was recorded. A new stranger 2 mouse (10–12-week-old male) was introduced to the cage containing an inanimate object before. The test mouse spent another 10 min in exploring the entire apparatus and time spent in each chamber was recorded.

### Motor, anxiety-, and depression-related behavioral tests

Open field: Locomotor activity was assessed over 10 min in an open field apparatus (38 cm × 38 cm × 38 cm). Locomotor activity was evaluated through total distance traveled by each mouse.

Elevated plus maze: The elevated plus maze (EPM) was comprised of two close arms (26 cm × 7 cm) and two open arms (26 cm × 7 cm) elevated 50 cm above the floor. Mice were placed in the center facing close arms and allowed to explore for 10 min. Anxiety-like behavior was assessed by time traveled in the open arms.

Tail suspension: Mice were gently suspended by the tail. Depression behavior was assessed over 6 min and evaluated through time of immobility except for respiration.

### Memory-related tests

Novel-object recognition: Novel-object recognition assay was carried out as described previously^16–18^. Two identical cubes were placed at the right and left corners of the apparatus. Mice were placed at the mid-point of the wall opposite the objects. After exposure for 10 min, mice were remove from the apparatus and have a rest for 1 h. One familiar object was placed at one corner of the apparatus, and one novel object was placed at the other corner. The animals were placed back into the apparatus and the duration of direct contact with two objects was recorded.

Social transmission of food preference (STFP): STFP assay was performed as described previously^17,18^. In this test, each mouse (observer) was fasted for 16 h in a single cage before test. Another female mouse, unfamiliar with the “observer”, was fed with cocaine for 30 min as “demonstrator”. After 30 min interaction time between “observer” and “demonstrator”, “observers” were fed with two types of food containing cocaine or cinnamon. The consumption of cocaine- or cinnamon-containing food was measured to test the memory ability. Normal mice preferred eating food containing cocaine instead of cinnamon because of the memory of the scent.

### Reduced representation bisulfite sequencing

Reduced representation bisulfite sequencing (RRBS) libraries were generated according to the previous study^19^. Genomic DNA (200 ng) was digested by *Msp* I to generate short fragments in a methylation-insensitive manner. After the digested DNA fragments were end-repaired and A-tailed, they were ligated to methylated Illumina adapters using Truseq DNA Sample Prep Kit (Illumina) following the user manual. The ligation products at the range of 150–700 bp were selected to perform bisulfite conversion using MethylCode™ Bisulfite Conversion Kit (Life Technologies). Conversion products were PCR-amplified using uracil + PCR polymerase (Zymo). The libraries were sequenced on an Illumina Hiseq X Ten platform under control of Hiseq Control Software.

### Primary neuron isolation and culture

Hippocampus were dissected from E16 mouse embryos in DMEM-HG (HyClone) at 4 °C. After removal of all the meninges, the tissues were digested with the digest medium (1 mg/ml papain, 5 mM EDTA, DNase Ι 0.1 mg/ml) at 37 °C for 30 min. The supernatant was removed and the tissues were re-suspended in the digest medium. After stop the reaction with FBS, cells were plated onto poly-D-lysine-coated plates. After culturing with plating medium (Neurobasal® Medium, 5% FBS, Supplement B27, GlutaMax Ι, Gentamycin) for 6 h, the cells were cultured in the culture medium (Neurobasal® Medium, Supplement B27, GlutaMax Ι, Gentamycin).

### Preparation of adeno-associated virus

AAV-DNMT3A-N-dCas9 (AAV9, 1.38 × 10^13^ genomic copies per ml), AAV-AAV-C-dCas9 (AAV9, 1.68 × 10^13^ genomic copies per ml) and AAV-5U6-sgRNAs-EGFP (AAV9, 1.31 × 10^13^ genomic copies per ml) were packaged and provided by Shanghai Taiting Biological Co., Ltd.

### Viral infection of mice

Mice were infected with appropriate rAAVs cocktails according to guidelines of IACUC. Briefly, mice were anesthetized by intraperitoneal injection of 5% chloral hydrate (200 μl /20 g body weight), and mounted in a stereotaxic holder in order to fix the skulls. To infect mouse brain, rAAVs mixtures (equal amount of AAV-DNMT3A-N-dCas9, AAV- AAV-C-dCas9 and AAV-5U6-sgRNAs-EGFP) were delivered by a Nanoject II (Drummond) system. 500 nl of mixtures were injected into the hippocampus (AP: −1.8 mm, ML: ± 1.5 mm, DV: −1.5 mm and −2 mm).

### Quantification and statistical analysis

Statistical analysis was performed using Graph Pad PRISM 7. Data are shown as the mean ± s.e.m. unless otherwise stated. No statistical methods were used to predetermine sample size. Experimenters were blind during all behavioral tests.

## Results

### Targeted DNA methylation at the *Mecp2* promoter in Neuro-2a cells

**Fig. 1:**
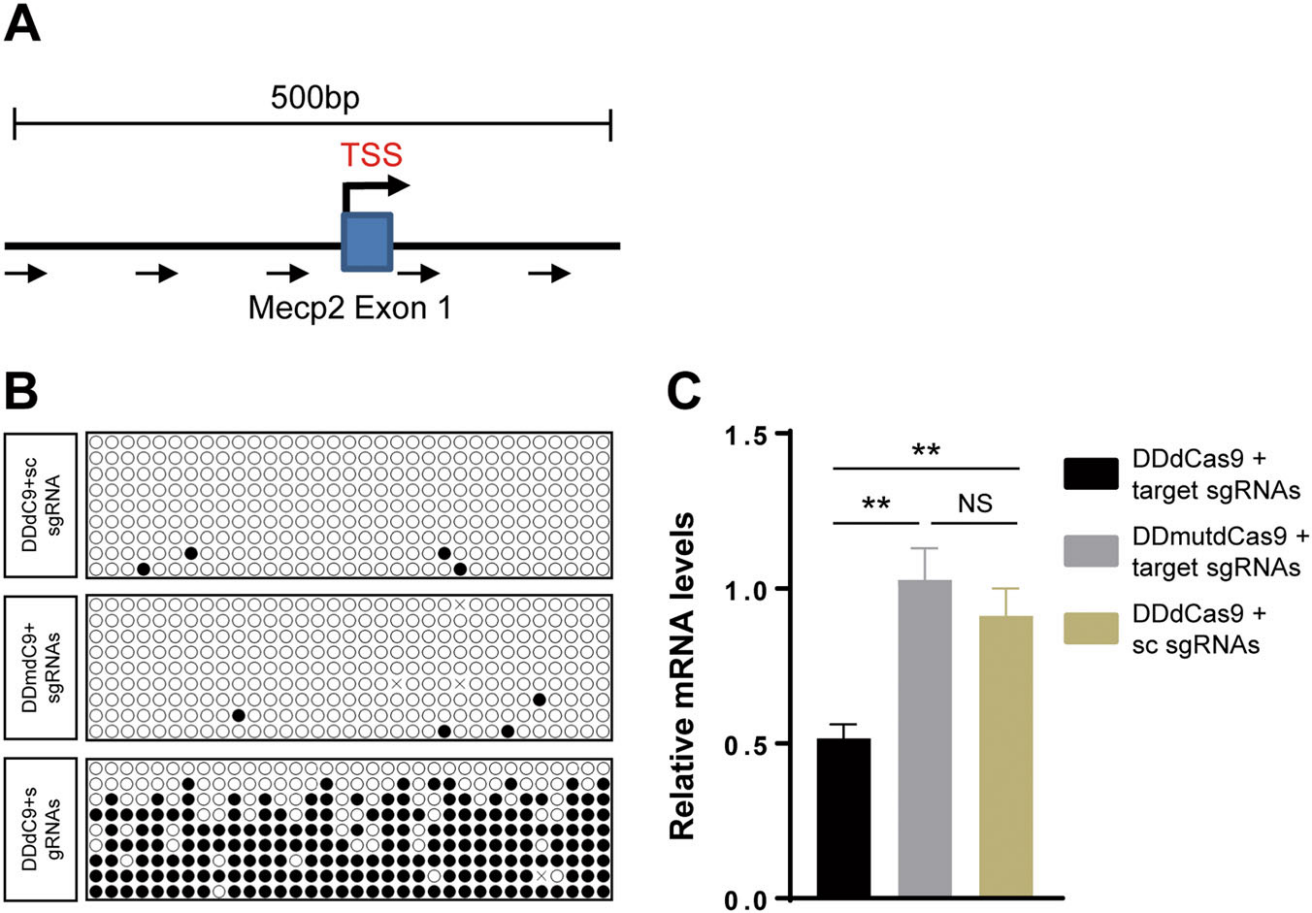
Targeted DNA methylation of Mecp2 promoter in N-2a cells using DNMT3L-DNMT3A-dCas9. **a** Graphical representation of the *Mecp2* locus showing sgRNAs target location. Transcription Start Site (TSS) is indicated by black arrow. The location of targets (1–5) for sgRNAs (Mecp2_1–5) are indicated by blue arrows. The Mecp2 exon is indicated by blue rectangle. **b** Representative methylation pattern as analyzed by bisulfite sequencing. **c** Targeted DNA methylation of *Mecp2* promoter led to downregulation of *Mecp2* expression in N2a cells. Expression of *Mecp2* was calculated by quantitative PCR at 4 days after transfection. DDdCas9, DNMT3L-DNMT3A-dCas9; DDmutdCas9, DNMT3L-DNMT3Amut-dCas9; sc sgRNA, scrambled sgRNA. Data were statistically analyzed by Student’s *t* test (**p < 0.01; NS, no significance), and shown as the mean ± s.e.m. (*n* = 3 from three independent experiments).

To study the role of *MECP2* promoter methylation in the ASD pathogenesis, we utilized a dCas9-based DNA methylation-editing tool to specifically increase methylation at the transcriptional start site (TSS) of *Mecp2*. To enhance the targeting efficiency, we fused the catalytic domain of DNA methyltransferase DNMT3A (DNMT3ACD) and its co-factor DNMT3L in tandem to the N-terminus of dCas9^20^. A catalytic dead DNMT3ACD mutant was used as a control. We also constructed a multi-sgRNA vector that simultaneously expresses five sgRNAs targeting the ~500 bp TSS region of *Mecp2* (Fig. 1a). All sgRNAs were demonstrated to be active as they can induce site-specific DNA cleavage with CRISPR-Cas9 system (Fig. S1A, B).

To examine the targeted methylation of *Mecp2* promoter, we transfected Neuro-2a (N2a) cells with DNMT3L-DNMT3A-dCas9 and multi-sgRNA vectors (referred hereafter as “methylation vectors”). At the same time, DNMT3L-DNMT3Amut-dCas9/multi-sgRNAs (“control vectors-1”) and DNMT3L-DNMT3A-dCas9/scramble-sgRNA (“control vectors-2”) were used as two negative controls. The transfected cells were selected with 2 μg/ml puromycin and 20 μg/ml blasticidin for 2 days. The surviving cells were harvested to prepare for genomic DNA, and DNA methylation was analyzed by bisulfite sequencing. As expected, the overall methylation level at the *Mecp2* promoter was up-regulated to about 65% with the transfection of “methylation vectors”, while the methylation level of this region was barely detectable with the treatment of “control vectors-1” or “control vectors-2” (Fig. 1b). Furthermore, *Mecp2* expression was significantly down-regulated as analyzed by qPCR (Fig. 1c).

To analyze the potential off-target effects, we collected N2a cells at 48 h after transfection and performed reduced representation bisulfite sequencing (RRBS) analysis. We found that the global DNA methylation patterns, total methylation level, and methylation proportion were similar between the methylation and control groups (Fig. S2).

Furthermore, we measured the DNA methylation levels of 131 predicted off-target sites using bisulfite sequencing (Table S1). Only 4 out of 131 regions showed slightly higher levels of DNA methylation in the methylation group as compared to the controls (Table S1). Hence, our system represents a precise tool for locus-specific DNA methylation.

### Targeted *Mecp2* promoter methylation down-regulates *Mecp2* expression in mice

**Fig. 2:**
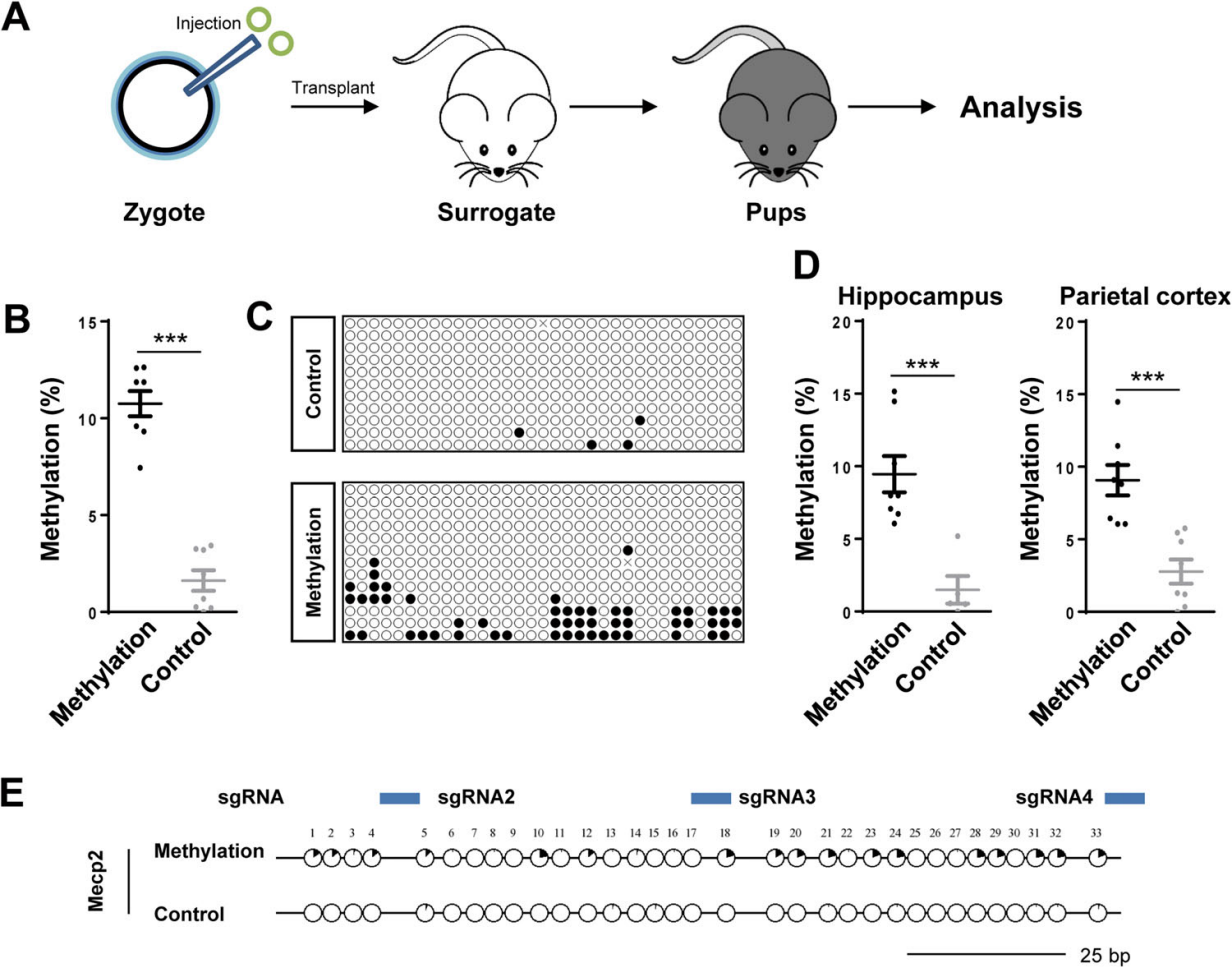
Targeted DNA Methylation of Mecp2 in vivo Using DNMT3L-DNMT3A-dCas9. **a** Schematic illustration of experimental procedure for targeted DNA methylation in vivo. Constructs expressing DNMT3L-DNMT3A-dCas9 or DNMT3L-DNMT3Amut-dCas9 along with sgRNAs targeting the *Mecp2* TSS region were pooled together and injected into mouse zygotes as indicated. **b** DNA methylation of targeted regions in the tail of male mice was analyzed by bisulfite sequencing. Each dot indicates one individual mouse. *n* = 8 mice/group; Data were statistically analyzed by Student’s *t* test (***p < 0.001), and shown as the mean ± s.e.m. **c** Representative methylation pattern in the tails of male mice as determined by bisulfite sequencing. **d** DNA methylation of target regions in the hippocampus (left) and parietal cortex (right) of male mice were analyzed by bisulfite sequencing. Each dot indicates one individual mouse. *n* = 8 mice/group; Data were statistically analyzed by Student’s *t* test (***p < 0.001), and shown as the mean ± s.e.m. **e** Methylation in the tail of male mice. Black portion of the circles indicates the methylation ratio in each CpG site. The results include all the sequencing data from mice described in (**b**). The *Mecp2* sgRNAs (2–4) are indicated by blue rectangles.

For targeted methylation of *Mecp2* promoter in vivo, the “methylation vectors” or “control vectors-1” were injected into the mouse zygotes (Fig. 2a). For the methylation group, we transferred 260 injected zygotes into 12 surrogate female mice, and obtained 61 live births (38 males and 23 females) (Table S2). For the control group, 248 injected zygotes were transferred into 10 surrogate female mice, and 65 live births (37 males and 28 females) were obtained (Table S2).

The genomic DNA from the tails of 3-week-old mice were subjected to bisulfate sequencing for analyzing the efficiency of the targeted DNA methylation. We found that male mice in the control group displayed less than 5% methylation at the *Mecp2* promoter (Fig. 2b, c). By contrast, the males from the methylation group showed a significantly higher methylation at the target locus (Fig. 2b, c, e). As *Mecp2* is an X-linked gene, the basal DNA methylation in this region is quite high in female mice. There was no significant difference in the DNA methylation in females from control and methylation groups (Fig. S3).

We also determined the levels of targeted DNA methylation in a variety of tissues. As shown in Fig. 2d, the DNA methylation levels in the hippocampus and parietal cortex were significantly higher in the methylation group relative to the control group. (hippocampus: 9.5 ± 3.0% vs. 1.5 ± 2.6%; parietal cortex: 9.1 ± 2.5% vs. 2.8% ± 2.0%; *p* < 0.001, unpaired *t* test). In addition, the DNA methylation levels were also significantly elevated in the heart, liver, kidney, lung, and testis of mice from the methylation group (Fig. S4).

**Fig. 3:**
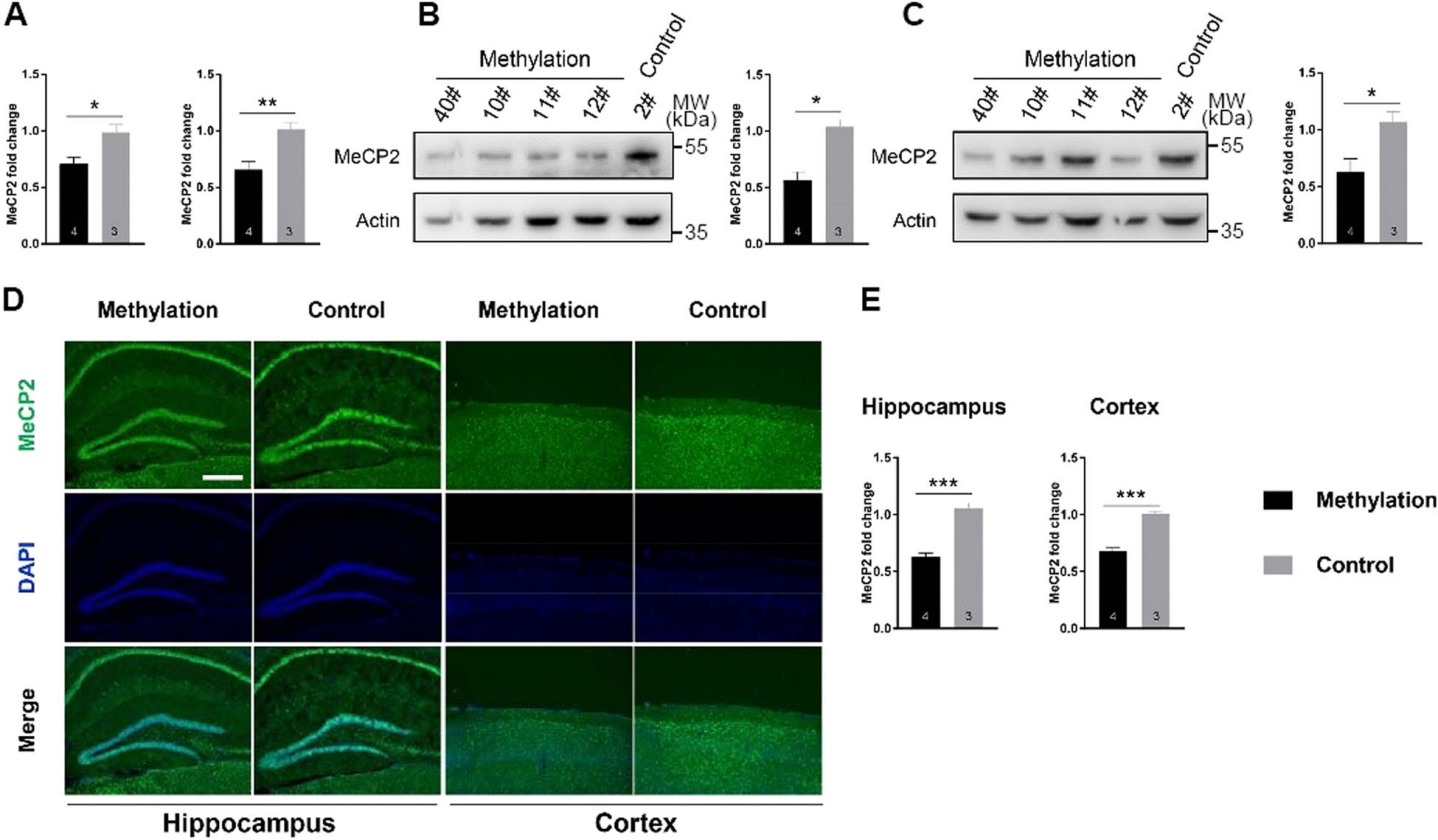
Targeted DNA methylation of Mecp2 decreased the Mecp2 expression. **a** Expression of *Mecp2* in the hippocampus (left) and parietal cortex (right) of 18-week-old mice as analyzed with quantitative PCR. Methylation *n* = 4, control *n* = 3. **b**, **c** Western blot analysis and quantification of MeCP2 expression in hippocampus (**b**) and parietal cortex (**c**). Methylation *n* = 4, control *n* = 3. **d** Representative fluorescence images of MeCP2 (green) in the hippocampus and parietal cortex. The nuclei were co-stained with DAPI (blue). Scale bar, 100 µm. **e** Expression of MeCP2 was calculated by fluorescence intensity from 7 samples and 14 slides. Left: MeCP2 expression in the hippocampus; Right: MeCP2 expression in the parietal cortex. Methylation *n* = 4, control *n* = 3; Data were statistically analyzed by Student’s *t* test (*p < 0.05; **p < 0.01; ***p < 0.001), and shown as the mean ± s.e.m. The number of animals was indicated in the respective columns.

Consistent with the data from N2a cells, the transcription of *Mecp2* in the hippocampus and parietal cortex was significantly reduced in male mice from the methylation group, as compared with the control group (Fig. 3a). Furthermore, Western blot analysis showed significantly lower MeCP2 confirmed by the weak immunofluorescence staining of MeCP2 protein in tissue sections as compared to the control male mice (Fig. 3d, e).

### Targeted DNA methylation at *Mecp2* promoter causes autism-like behaviors

**Fig. 4:**
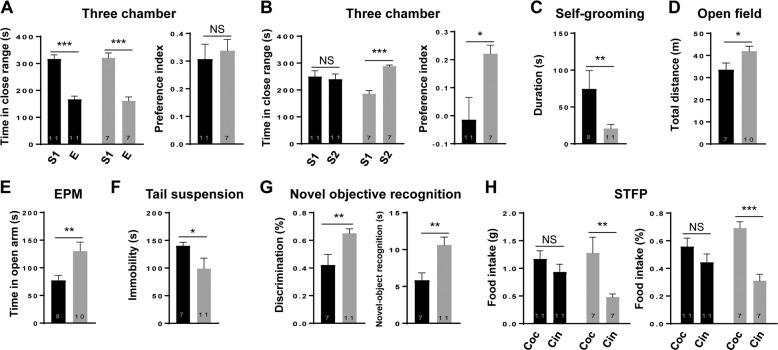
Targeted DNA methylation of *Mecp2* caused ASD-Like phenotypes in mice. **a**, **b** Social behavior as examined by the three-chamber test. (**a**) Left: the time of mice interacting with either the stranger mouse (S) or the empty cage (E) in the second phase of the three-chamber test. Right: Data are ratios of interaction time with a stranger mouse to interaction time with an empty cage. (**b**) Left: the time of mice interacting with either the familiar mouse (S1) or the novel mouse (S2) in the third phase of the three-chamber test. Right: Data are ratios of interaction time with a stranger mouse to interaction time with a familiar mouse. Methylation *n* = 11, control *n* = 7. **c** Repetitive behaviors as measured by the total time on self-grooming over a 10 min period. Methylation *n* = 8, control *n* = 11. **d** Locomotor activity as examined by the total distance traveled in the open field. Methylation *n* = 7, control *n* = 10. **e** Anxiety-related behavior as measured by the time in the open arms in the elevated plus-maze assay (EPM). Methylation *n* = 8, control *n* = 10. **f** Depression-related behavior as measured by the immobility time in the tail suspension assay. Methylation *n* = 7, control *n* = 11. **g** Memory-related behavior as measured by the total time spent on interacting with novel object and familiar object over a 10 min period in object recognition assay. Right: Discrimination ratio (novel-object interaction/total interaction with both objects); Left: novel-object recognition (novel-object interaction–familiar object interaction). Methylation *n* = 7, control *n* = 11. **h** Social memory-related behavior measured by the total consumption (left) and consumption ratio (right) of cocaine and cinnamon eaten by the male observer mice in the social transmission of food preference assay (STFP). Coc, cocaine; Cin, cinnamon. Methylation *n* = 11, control *n* = 7. Data were shown as the mean ± s.e.m, the number of mouse were indicated in respective bars. Methylation male mice were indicated in black and control male mice were indicated in gray. Statistical analysis is performed by Student’s t test (NS, *p* > 0.05; *p < 0.05; **p < 0.01; and ***p < 0.001).

It is well known that abnormal *MECP2* expression leads to ASD in human and ASD-like behaviors in mice^11,21^. In order to investigate whether manipulation of DNA methylation at the *Mecp2* promoter will cause abnormal behaviors in mice, we first examined the social interaction by using the three-chamber test. Prior to the social interaction, each mouse showed no preference in the chambers (data not shown). We found that mice from both methylation and control groups showed similar preference to the mouse over an object (Fig. 4a). When facing a familiar mouse versus an unfamiliar mouse, mice from the control group preferred to explore the unfamiliar mice, whereas mice from the methylation group showed no preference between familiar and unfamiliar mice (Fig. 4b). This result indicates that mice from the methylation group displayed a reduction in conspecific recognition or in their interest for social novelty compared with the control mice. Furthermore, we found that mice from the methylation group spent significantly longer time in self-grooming (Fig. 4c), reflecting an increased repetitive behavior.

Rett syndrome patients with *MECP2* mutation also display reduced locomotion and anxiety^11,22^. We thus examined the locomotor activity of the mice by the open field test. We found that mice from the methylation group traveled a shorter total distance in the open field as compared to the control mice (Fig. 4d). Furthermore, mice from the methylation group had higher anxiety as they spent significantly less time in the open arms as compared to the control mice in the elevated plus-maze (EPM) test (Fig. 4e). In a tail suspension test, mice from the methylation group exhibited higher immobility time as compared to the control mice (Fig. 4f), indicating an elevated depression-like behavior in these mice.

Finally, we performed a series of behavioral tests to examine the memory- and cognition-related behaviors. In the novel-object recognition test, mice in the methylation group spent less time playing with a novel object relative to a familiar one than the control mice (Fig. 4g), suggesting deficiency in memory retention or reduced novelty seeking. Furthermore, the social transmission of food preference (STFP) test showed that mice of the methylation group failed to acquire the food preference of another mouse following 30-min co-housing in the absence of the food (Fig. 4h). Taken together, our data indicated that promoter-specific DNA methylation of *Mecp2* gene could lead to behavioral alterations similar to those caused by *Mecp2* deficiency.

### *Mecp2* promoter methylation in the hippocampus leads to defective social behaviors

**Fig. 5:**
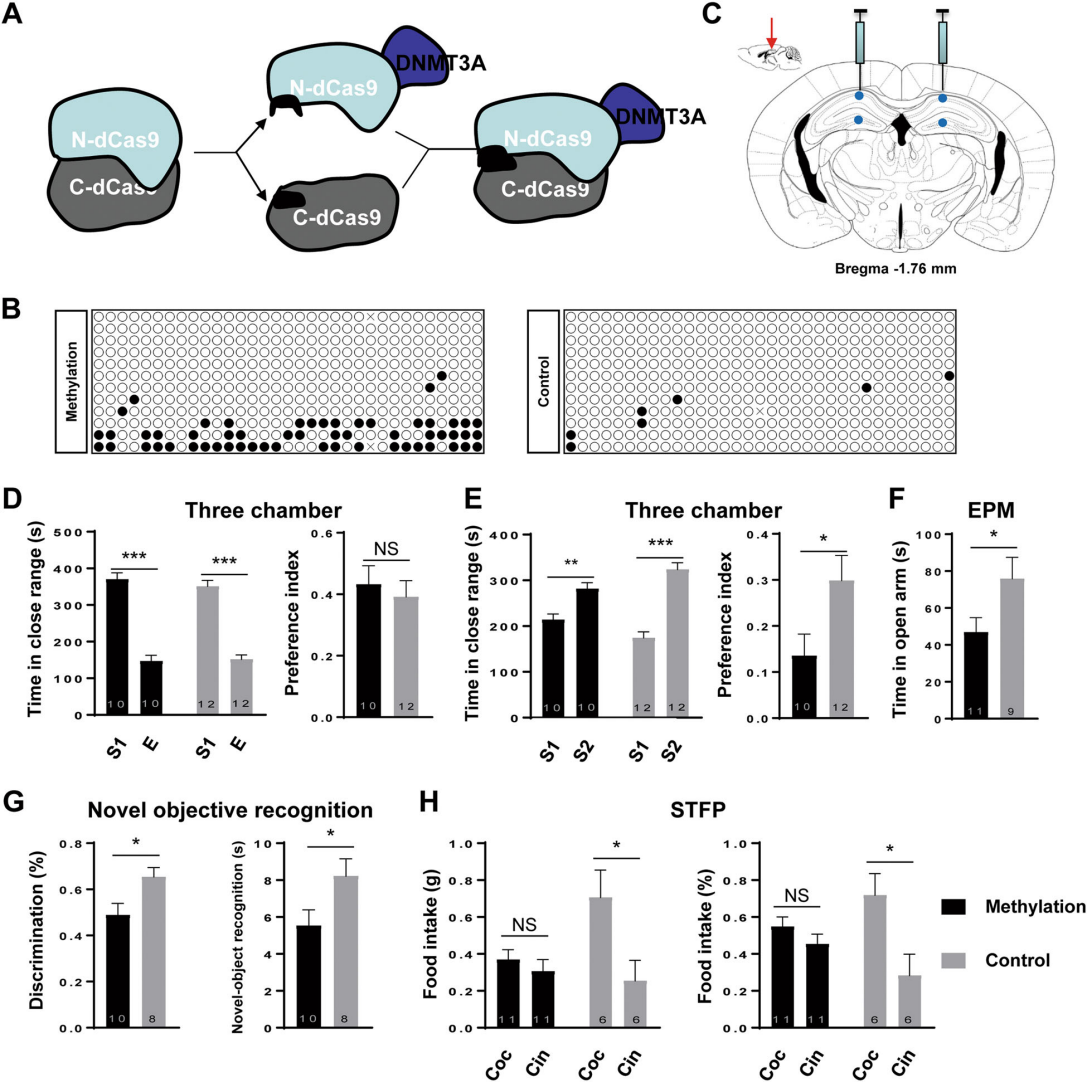
Targeted DNA methylation of *Mecp2* in the hippocampus by microinjection of AAVs. **a** Schematic illustration of structure and assembly of AAV-DNMT3A-split-dCas9 system. **b** Representative methylation pattern as determined by bisulfite sequencing in cultured neurons infected with AAV-DNMT3A-N-dCas9, AAV-C-dCas9 and AAV-5U6-sgRNAs (methylation) or AAV-5U6-sgRNAs only (control). **c** Schematic illustration of viral injection point. The red arrow indicates the injection direction; the blue dots indicate the injected points. **d**, **e** Social behavior as examined by the three-chamber test. **d** Left: Time of mice interacting with either the stranger mouse (S) or the empty cage (E) in the second phase. Right: The ratio of interaction time with a stranger mouse to interaction time with an empty cage. (E) Left: Time of mice interacting with either the unfamiliar mice (S2) or the familiar mice (S1) in the third phase. Right: The ratio of interaction time with an unfamiliar mouse (S2) to a familiar mouse (S1). Methylation *n* = 10, control *n* = 12. **f** Anxiety-related behavior as measured by the time in the open arms in the elevated plus-maze assay (EPM). Methylation *n* = 11, control *n* = 9. **g** Memory-related behavior as measured by the total time spent on interacting with novel-object recognition (right) and discrimination ratio (left) over a 10 min period in object recognition assay. Methylation *n* = 10, control *n* = 8. **h** Social memory-related behavior measured by the total consumption (left) and consumption ratio (right) of cocaine and cinnamon eaten by the male observer mice in the social transmission of food preference assay. Coc, cocaine; Cin, cinnamon. Methylation male mice were indicated in black and control male mice were indicated in gray. Methylation *n* = 11, control *n* = 16. Data are shown as the mean ± s.e.m, the number of mouse were indicated in respective bars. Statistical analysis was performed by Student’s *t* test (NS, *p* > 0.05; *p < 0.05; **p < 0.01; and ***p < 0.001).

To determine whether *Mecp2* promoter methylation in the brain is sufficient to alter the behaviors of mouse, we set out to perform epigenetic modification in specific brain regions. To this end, we developed the AAV-based methylation vectors that target the TSS of *Mecp2* gene (Fig. 5a). Neurons infected with these viruses showed a significantly higher level of methylation in *Mecp2* TSS region (~14 %) as compared to neurons infected with the control vector (~1%) (Fig. 5b).

Given the importance of the hippocampus in memory and cognitive behaviors, AAV-based methylation vectors were bilaterally injected into the hippocampus of adult mice (Fig. 5c). The infection efficiency was verified in brain sections at two weeks after injection, and all behavioral tests were then performed at 4 weeks after injection. As shown in Fig. 5d–h, we found similar abnormality in social interaction in three-chamber test, increased anxiety on elevated open arm, novel-object recognition, and acquisition of food preference in STFP test, although the degrees of abnormality were lower than those found for mice with zygote-based methylation targeting. By contrast, we found no significant abnormality in locomotive and grooming activities, and depression-like behavior (Fig. S5). These results support the notion that specific methylation of *Mecp2* promoter in the brain could lead to ASD-like behaviors.

## Discussion

In this study, we demonstrated a critical role of *Mecp2* promotor methylation in the ASD pathogenesis, by locus-specific methylation of *Mecp2* promotor using an improved dCas9-based methylation targeting method. Methylation of *Mecp2* promotor led to reduction in *Mecp2* gene expression, and alteration of a panel of behaviors mimicking the ASD symptoms. We further showed that specifically increasing the *Mecp2* promoter methylation in the hippocampus was sufficient to induce most of the behavioral changes.

DNA methylation on cytosine of a gene promoter usually leads to the silencing of gene expression. Abnormal DNA methylation has been observed in cancer and neurological disorders^23^. The functional significance of DNA methylation has been exploited by using transgenic mice with disruption of specific genes involved in the DNA (de)methylation, or DNMTs inhibitors, such as azacitidine and decitabine. However, such approach can only be applied non-selectively at the global genome level, but not at specific loci^23^.

The recently developed genome-modifying tools, such as zinc fingers (ZFs), transcription activator-like effectors (TALEs), or the CRISPR/Cas9 system have been successfully applied to loci-specific epigenome editing^23,24^. Several groups successfully repurposing the CRISPR-Cas9 system for targeted DNA methylation^25–30^. In this study, we further improved the targeting efficiency of the dCas9-based vector by adding a sequence encoding DNMT3L, which functions as a co-factor of DNMT3A to enhance DNA methylation. As a result, the locus-specific methylation of *Mecp2* promotor down-regulated *Mecp2* expression, and mice with *Mecp2* promoter methylation exhibited ASD-like phenotypes. Our study thus provides the first evidence to support the causative relationship between DNA methylation and ASD-like phenotypes.

Most of the behaviors from mice with Mecp2 promoter methylation were in agreement with previous findings from Mecp2-deficient mice. However, there are contradict data in the anxiety-related behaviors by using different strains of Mecp2-deficient mice^24,31–33^. In our study, increasing Mecp2 promoter methylation led to elevated anxiety levels. Furthermore, hippocampus-specific Mecp2 promoter methylation also induced increased anxiety, reduced social interaction, and defective memory, which is consistent with the results from hippocampus-specific Mecp2 knockout mice^34^. Nevertheless, no significant abnormality in locomotive and grooming activities, and depression-like behavior were observed in the mice with hippocampus-specific *Mecp2* promoter methylation, implying these behaviors may be related to other brain areas other than hippocampus.

Loci-specific epigenome editing not only help understand the causative relationship between DNA methylation in a specific DNA loci and pathological situations, but also have potential therapeutic applications. Indeed, Choudhury et al. used dCas9-TET1 fusion to selectively de-methylate targeted regions within BRCA1 promoter as directed by the designed single-guide RNAs (sgRNA), leading to the transcriptional up-regulation of the gene^35^. Recently, Liu et al. demonstrated that a locus-specific demethylation of Fragile X mental retardation (*FMR1*) gene, a putative fragile X syndrome (FXS)-causative gene, could correct the phenotype of FXS patient-derived induced pluripotent stem cells^30^. Xu et al. developed a high-fidelity CRISPR/Cas9-based gene-specific dioxygenase to rescue gene expression in vitro and attenuate renal fibrosis in vivo^36^. In this study, by targeted editing of DNA methylation, we demonstrated that methylation at the *Mecp2* promoter is sufficient to down-regulated *Mecp2* expression and induce autism-like behaviors in mice. Further, *Mecp2* methylation specifically in the hippocampus is able to induce most of the behavioral alteration. Our finding therefore demonstrated DNA methylation of *Mecp2* promoter plays a causative role in autism-related symptoms, warranted potential drug discovery targeting such epigenetic alteration.

## Supplementary information

Supplementary Figure legends

Fig S1

Fig S2

Fig S3

Fig S4

Fig S5

Table S1

Table S2

Table S3

## Data Availability

High-throughput sequencing data have been deposited in the NCBI Sequence Read Archive database with an accession code (SRP234617). Plasmids were deposited in Addgene with following catalog numbers: pGL3-U6-sgRNA (Addgene, 51133), pSt1374-N-NLS-DNMT3L-L-DNMT3A-Ldcas9-NLS (Addgene, 112209), pSt1374-N-NLS-DNMT3L-L-DNMT3Amut-L-dcas9-NLS (Addgene, 112210), pAAV-CAG-C-intein-C-spC9-H840AN863A-2xNLS-hGH (Addgene, 112211), pAAV-CAG-NLS-DNMT3A-L-N-spC9-Nintein-hG (Addgene, 112212), pAAV-5U6- sgRNAs-hsyn-EGFP (Addgene, 112213), pSt1374m-EF1alpha-N-NLS-DNMT3L-LDNMT3A-L-dcas9 -NLS (Addgene, 112214) and pSt1374m-EF1alpha-N-NLS-DNMT3L-L-DNMT3Amut-L-dcas9-NLS (Addgene, 112215).
