# Supplementary material for: Locus-specific DNA methylation of Mecp2 promoter leads to autism-like phenotypes in mice
Source: Cell Death Dis. 2020 Feb 3;11(2):85. doi: 10.1038/s41419-020-2290-x (PMC6997184; doi:10.1038/s41419-020-2290-x)
Supplement: Supplementary file 1 — Supplementary Figure legends [file 41419_2020_2290_MOESM1_ESM.docx]

**Supplementary Figure Legends**

**Fig S1** The Cas9/sgRNAs mediated modifications of mouse *Mecp2* locus. **(A)** Detection of Cas9/sgRNAs-mediated cleavage of TSS region of Mecp2 by T7EN1 cleavage assay. M, DNA marker. sg1, sgRNA 1; sg2, sgRNA 2; sg3, sgRNA 3; sg4, sgRNA 4; sg5, sgRNA 5. Con, negative control. **(B)** Sequences of the modified target loci. TA clones of the PCR products were analyzed by DNA sequencing. The PAM sequences are underlined and highlighted in green; the targeting sequence is highlighted in red; deletions (-), insertions (+). N/N represents positive colonies out of total sequenced samples.

**Fig S2** The off-target effects detected by RRBS analysis. **(A)** Pearson’s correlation for all pair-wise comparisons of RRBS data. Numbers, correlation coefficient; histogram, distribution of % CpG methylation; blue and green pattern, methylation ratio density for pair-wise comparisons of RRBS data. **(B)** Genome-wide CpG methylation heat map using RRBS. About 42% of genome-wide CGIs (6845 out

of 16009) covered by all groups were analyzed. **(C)** Average DNA methylation level. Average DNA methylation level of each CpG, CHG and CHH covered more than 3 reads in genome. **(D)** Proportion of methylated C. The proportion of methylated cytosine sites, covered more than 3 reads in genome, in total CpG, CHG and CHH. **(E)** Representative off-target results of five sgRNAs. Methylation level (x axis) of 100bp region around target site at different potential off-target loci (y axis).

**Fig S3** DNA methylation at *Mecp2* promoter in female mice. (A) DNA methylation at Mecp2 promoter in WT female mice. (B) DNA methylation at Mecp2 promoter in female mice from treatment group. (C) DNA methylation at Mecp2 promoter in female mice from control group. (D) Summary of DNA methylation levels from A-C.

**Fig S4** Targeted DNA methylation of *Mecp2* in different tissues. DNA methylation of targeted methylation regions of different tissues of newborn male mice were analyzed by BSP. Methylation levels (Y axis) of MeCP2 locus among different group (X axis) were plotted. Each dot indicates one individual mouse. Data were statistically analyzed by Student’s t test, and shown as the mean ± s.e.m. Data were statistically analyzed by Student’s *t* test (*p < 0.05).

**Fig S5** Targeted DNA methylation of *Mecp2* in the hippocampus had no effect on the locomotor activity (A), repetitive behaviors (B) and depression-related behavior (C). (A) Locomotor activity was examined by quantifying the total distance of the methylation (black) and control (gray) male mice in the open field assay. Methylation n=9, control n=7. (B) Repetitive behaviors was measured by quantifying the total time spent grooming over a 10-min period of the methylation (black) and control (gray) male mice in the self-grooming assay. Methylation n=11, control n=8. (C) Depression-related behavior was measured by the immobility time of the methylation (black) and control (gray) male mice displayed in the tail suspension assay. Methylation n=11, control n=9. Data are shown as the mean ± s.e.m, the numbers of mice are indicated in bars. Statistical analysis was performed by Student’s t test (n.s., p > 0.05).
