# Supplementary figures and images for: Locus-specific DNA methylation of Mecp2 promoter leads to autism-like phenotypes in mice
Source: Cell Death Dis. 2020 Feb 3;11(2):85. doi: 10.1038/s41419-020-2290-x (PMC6997184; doi:10.1038/s41419-020-2290-x)

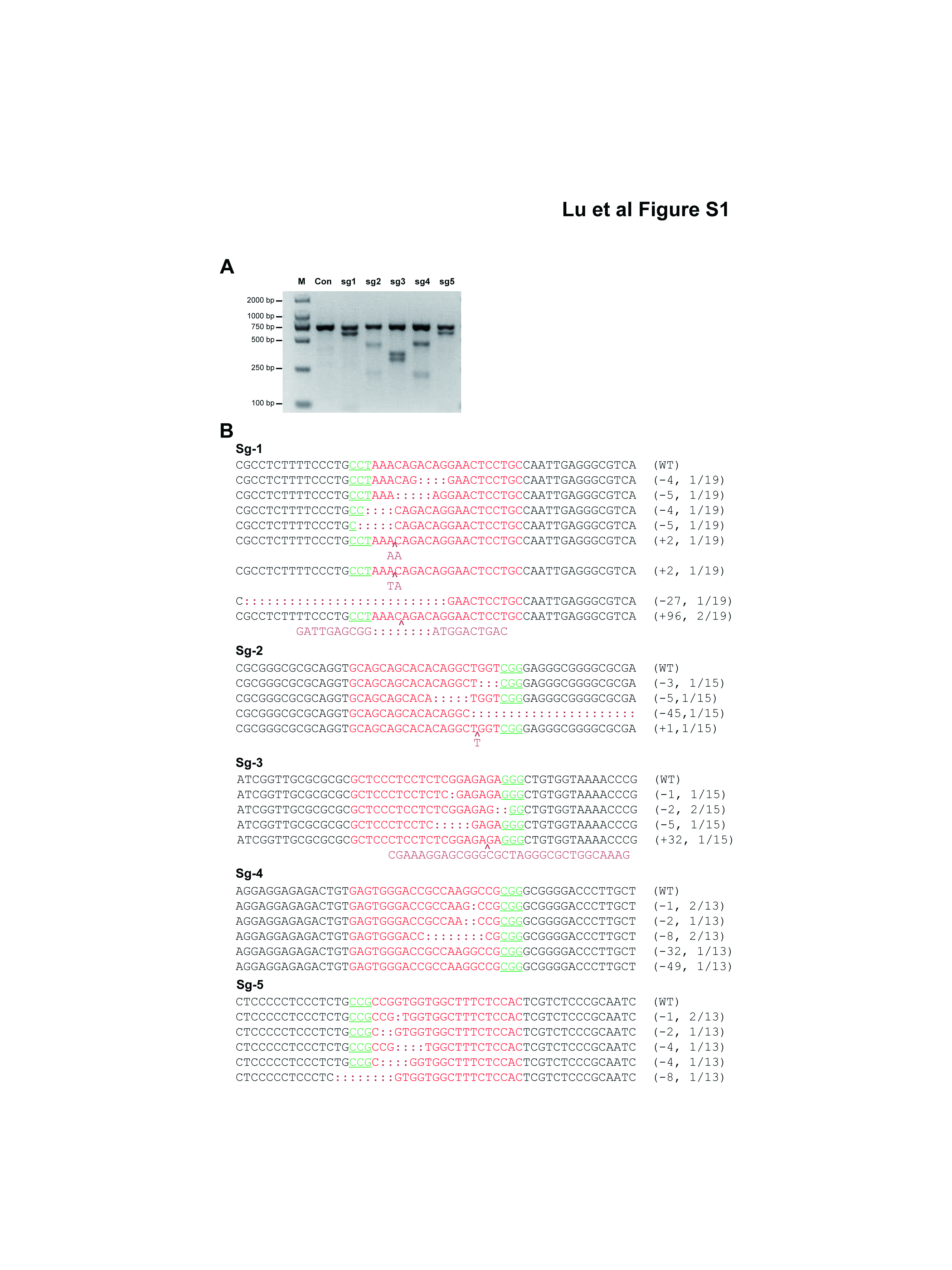

Supplement: Supplementary file 2 — Fig S1 [file 41419_2020_2290_MOESM2_ESM.tif]

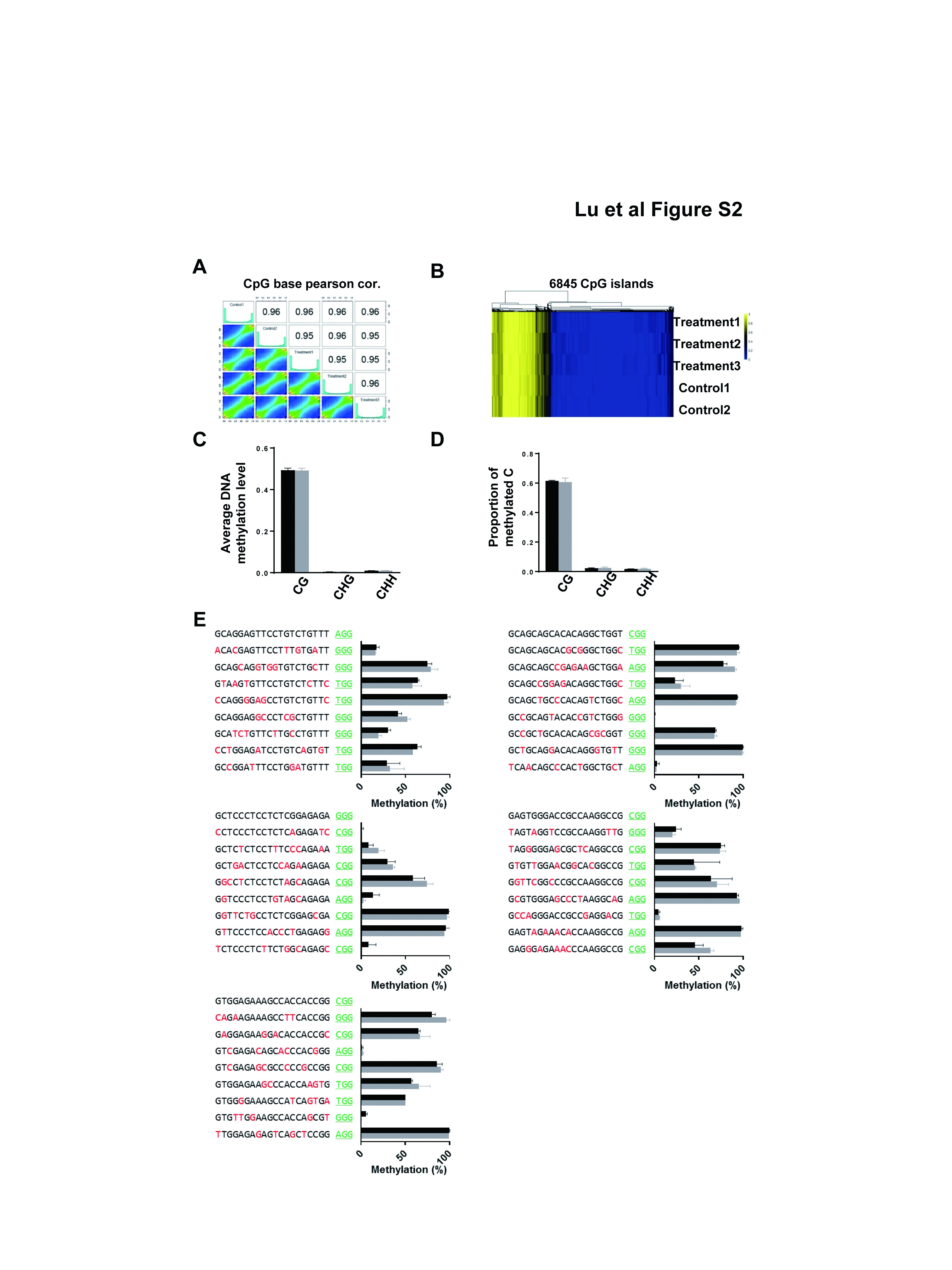

Supplement: Supplementary file 3 — Fig S2 [file 41419_2020_2290_MOESM3_ESM.tif]

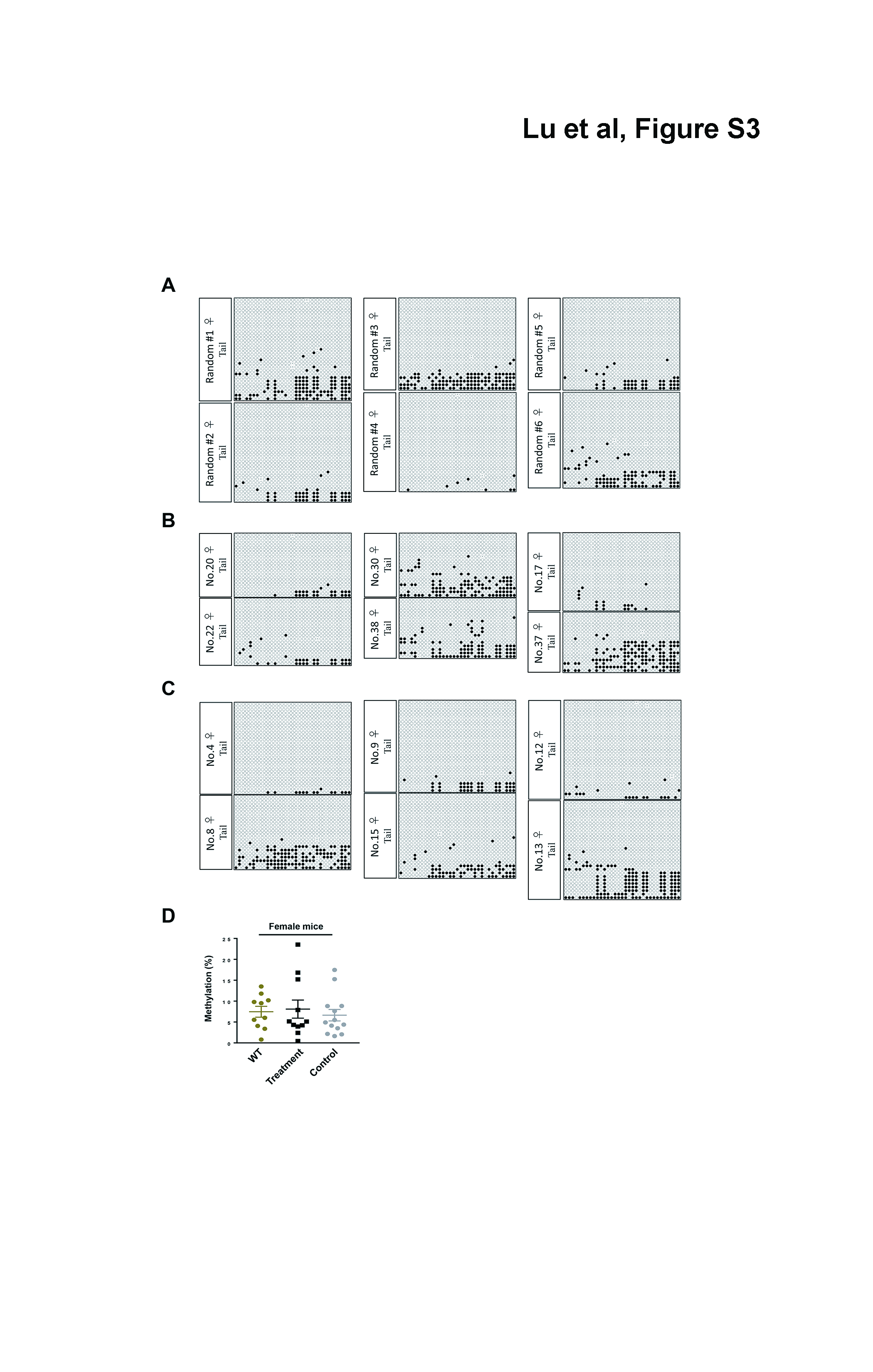

Supplement: Supplementary file 4 — Fig S3 [file 41419_2020_2290_MOESM4_ESM.tif]

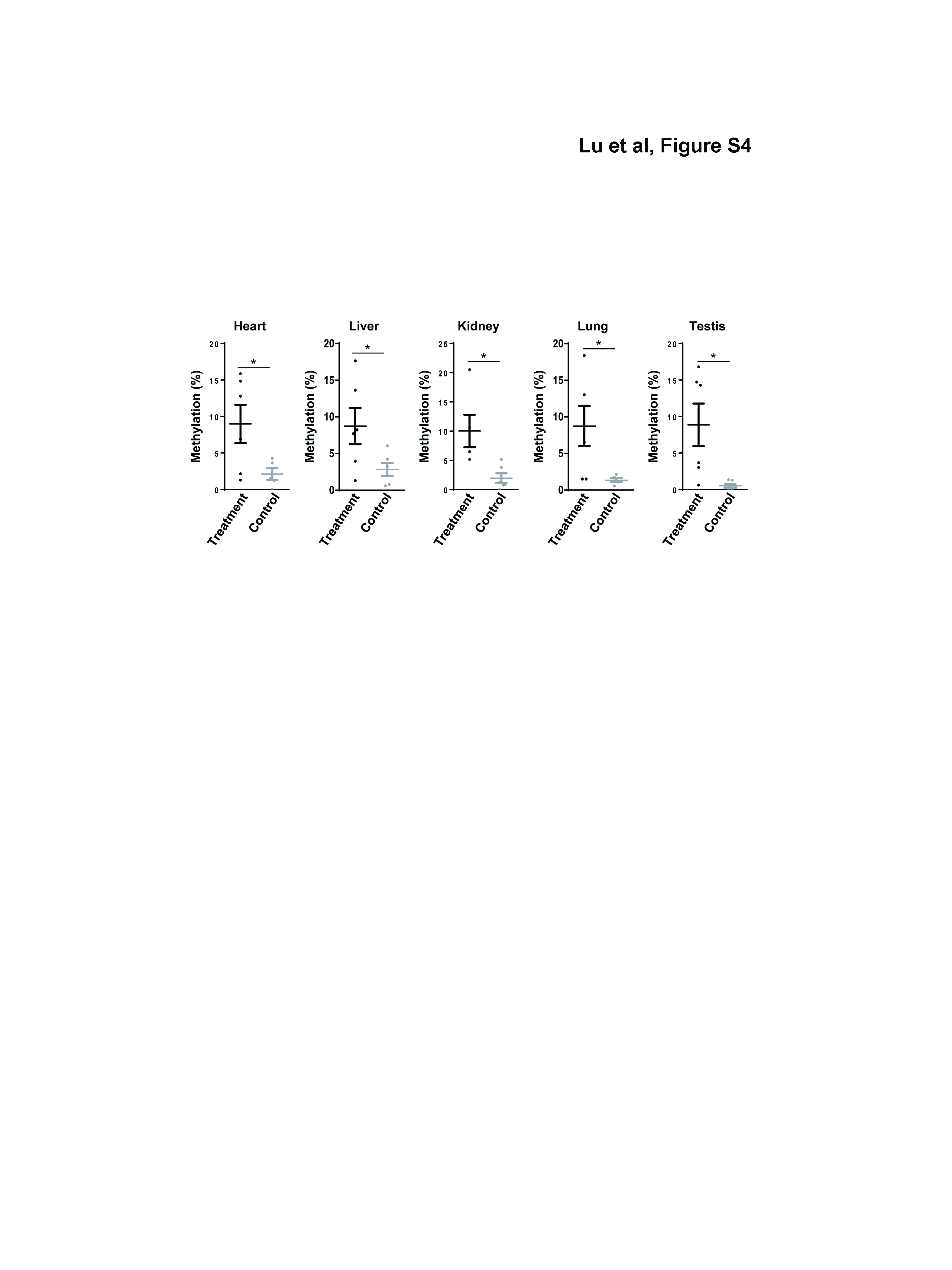

Supplement: Supplementary file 5 — Fig S4 [file 41419_2020_2290_MOESM5_ESM.tif]

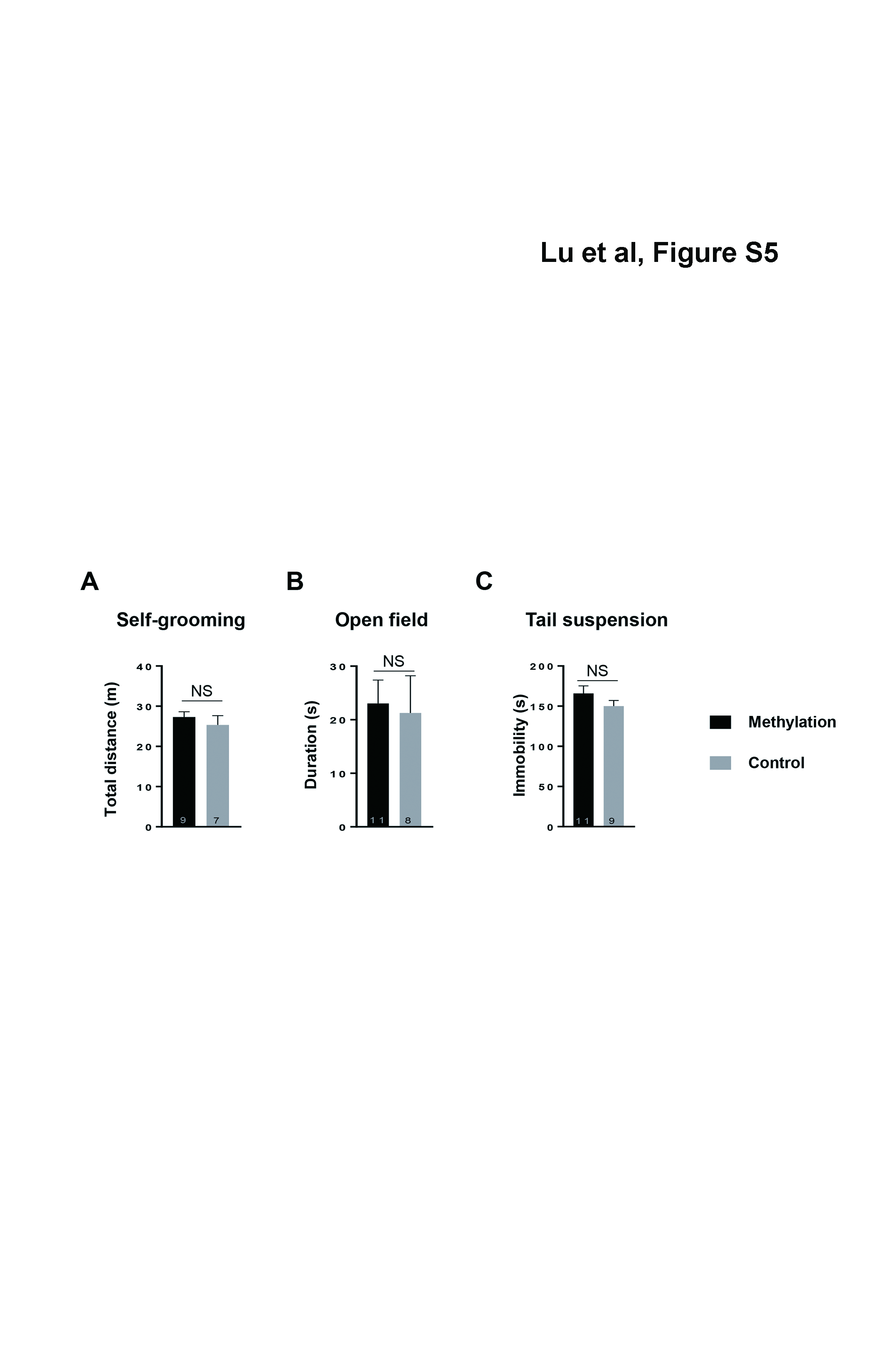

Supplement: Supplementary file 6 — Fig S5 [file 41419_2020_2290_MOESM6_ESM.tif]
